# The development of a framework for clinical education programme of undergraduate nursing students in Ghana

**DOI:** 10.1186/s12912-024-01915-y

**Published:** 2024-04-23

**Authors:** Gilbert Ti-enkawol Nachinab, Susan Jennifer Armstrong

**Affiliations:** 1 Department of Nursing Education, School of Therapeutic Sciences, University of the Witwatersrand, Johannesburg, South Africa; 2 Department of General Nursing, University for Development Studies, Tamale, Ghana

**Keywords:** Clinical education, Undergraduate, Nursing, Framework

## Abstract

**Introduction:**

Clinical education is an important aspect of the training of nursing students but it is faced with challenges in Ghana. The development of a framework will respond to the need for improvement in the quality of clinical nursing education. This study describes part of a larger study which culminated in the development of a framework for a clinical education programme for undergraduate nursing students in Ghana. The aim of the current study was to integrate findings from a scoping review and situational analysis to develop a framework for clinical education in nursing.

**Methods:**

A sequential multimethod design approach was used to conduct the study. A scoping review on the practices that facilitate clinical nursing education and situational analysis were first conducted. The lessons learnt from the scoping review and the situational analysis provided the data matrix that was triangulated to develop the framework. The framework was developed using the model for clinical education developed by South African Nursing Education Stakeholders in consultation with experts in nursing education. An implementation plan was developed from the framework and evaluated using a Delphi technique.

**Findings:**

The resulting framework indicates the need for effective communication and collaboration between nursing education institution and the service setting to ensure that there is a well-structured clinical placement, formal supervision system and effective clinical assessment of students. The framework also proposes that to ensure quality clinical nursing education there is the need for Nursing Education Institutions to implement innovative and cost-effective clinical teaching methods.

**Conclusion:**

The framework spells out the functions of the various stakeholders in nursing education and how these can be integrated and implemented to enhance quality clinical nursing education. Effectiveness of the thematic areas of the framework will increase the quality of clinical nursing education.

**Supplementary Information:**

The online version contains supplementary material available at 10.1186/s12912-024-01915-y.

## Introduction

Clinical nursing education involves all the real-life or practice-based teaching and learning activities that take place within the clinical learning environment [1]. The nursing profession is practice-oriented hence the training of nurses must be tailored towards the acquisition of practical competencies that will make graduates safe and quality practitioners [2–4]. The emphasis on clinical teaching and learning might have an influence on the role that clinical facilities play in nursing education. The location of the clinical facilities and the availability of resources are major factors in determining the quality of clinical education.

Nursing education has moved from a hospital-based apprenticeship system of training into higher education where the Nursing Education Institutions (NEIs) have more autonomy and responsibility [5]. Though undergraduate nursing programmes produce nurses with higher academic qualifications under this system, the ability of most universities to ensure effective clinical nursing education is challenging. Universities typically offer the undergraduate nursing programme in addition to other programmes of study and this might lead to competition for financial resources to meet the training needs of all the programmes. For instance, the nursing programme requires the NEI to provide resources not required by other programmes such as human resources to cover academic and clinical supervision, a skills laboratory, as well as a library, and information and communication technology facilities to enable students to access support whether in theoretical or clinical placements.

Nursing students’ capacity to learn clinical skills is influenced by the behaviour of their clinical instructors [6–8]. Clinical instructors’ abilities that positively influence the students’ learning process include communication skills, coaching and clinical nursing competence [6, 9–11]. These positive clinical instructors’ abilities can be obtained through working experience, further training and willingness to support students’ clinical learning.

Students on clinical placement often come to the ward with placement objectives that require support from the clinical nurses [12–14]. In some instances, students of different levels of their studies may be placed simultaneously in a clinical area which implies they need differing levels of support. The nurses in the clinical area often concentrate on rendering nursing care to patients, as this is their primary role, and may not be in a position to adequately support students to achieve their clinical placement objectives [12, 15]. Where there is no organized system of rendering support to students during clinical placement, achieving clinical placement objectives will be challenging.

Interactions with undergraduate nursing students have revealed that they face challenges during clinical placement. Lecturers, clinical nurses and students are all sceptical about how effective clinical placements are in enhancing skills acquisition among undergraduate students in Ghana. Studies have revealed that there is a gap between what students are taught in class and what they learn during clinical placements [15–18]. Registered nurses who act as clinical instructors may sometimes carry out procedures regardless of the standardised way in which students are taught in the class and this confuses students in their clinical learning process [15]. Successful experience with the clinical environment largely depends on the level of supervisory support from clinical supervisors and nurses [19].

Clinical instructors need some training to acquire competencies necessary for them to perform their roles. Clinical instructors are of the view that academic institutions should institute a course on clinical facilitation to give them formal training [20]. A comprehensive educational preparation will give the clinical instructors the teaching skills to enable them to stimulate clinical reasoning and professional development of students [21]. In Ghana, registered nurses generally act as clinical instructors since they are engaged in the clinical teaching of students in the clinical facilities. Nurses assume the role of clinical instructors out of their desire to help students. However, some nurses may not actively engage students in their clinical learning because they are not trained as clinical instructors or preceptors. Studies conducted in Ghana have indicated the need to build the capacity of nurses acting as clinical instructors so that they can meet the training needs of students during clinical placement [22, 23].

The number of nursing students in NEIs in each region of Ghana generally exceeds the clinical facilities available for clinical placement leading to overcrowding during clinical placement [22]. Overcrowding of students in the clinical area is a barrier to effective clinical nursing education. When students are crowded in the clinical area, they have difficulties in observing nursing procedures and getting opportunities to practice them themselves. Overcrowding of students also increases the burden on nurses who try to assist students to learn clinical skills.

Overcrowding of students does not only occur in the clinical facilities but in the skills laboratories in the Nursing Education Institutions. Effective skills training in the skills laboratory depends on the availability of equipment, space and proper scheduling of students to avoid overcrowding [24, 25]. The presence skills educators in the skills laboratory is also a factor that will enhance the teaching of kills. However, anecdotal evidence indicates that in Ghana nursing lecturers in most undergraduate nursing programmes focus on teaching the theoretical aspects their allocated courses.

Factors that predominantly influence the quality of clinical nursing education include student-related issues, nurse and faculty-related factors, management issues and availability of training facilities [26]. Students are the main focus of clinical teaching hence they need to for them to be self-motivated and confident [9]. Simulation is an essential component of clinical nursing education [27]. Simulation enables students to learn skills in an environment that resembles the clinical setting with no fear of causing harm to human life [28]. Some nursing education institutions use simulation to complement clinical teaching. However, a lack of space, equipment and clinical teaching staff may be a challenge in instituting simulation.

In response to the need for improvement in the various aspects of clinical education, several studies have been conducted in Ghana and recommendations made based on the findings for improvement [29–32]. Preceptorship which a major concept in clinical education have been researched and recommended for implementation in the Ghana to improve clinical education in nursing [31]. Also, a framework for assessing clinical competence of nursing students in Ghana have been recentedly developed [33]. In Ghana, as part of the accreditation process for NEIs, the NEIs are required to sign memoranda of understanding with clinical facilities to enable them have a smooth clinical placement of students. The existing studies all points to the need to improve the clinical education aspect of the Bachelor of Nursing programme in Ghana. Despite, all these existing recommendation to improve clinical education, there is the need to develop a framework that spells out the various components of clinical education in Ghana and how their functions can be integrated and implemented to enhance quality clinical nursing education. This study therefore aimed at developing a framework for clinical nursing education in Ghana which will be feasible and acceptable to all students, clinical skills lecturers and universities offering the degree in nursing programme.

## Materials and methods

### Study setting

The study was conducted at a public university in the northern Ghana and three hospitals where students from the university are placed for clinical experience. The university has seven [7] schools and seven [7] faculties. The School of Nursing and Midwifery was created in November 2020. The school of Nursing and Midwifery has six departments and offers Bachelor of Science degree in Nursing, Nurse Practitioner, Midwifery and Paediatric Nursing. The three hospitals are a district level hospital, a regional level hospital and a tertiary hospital. The tertiary hospital serves as a referral facility for all the health facilities within the northern zone of Ghana.

### Research design

The research design spells out the strategies adopted by the researcher to attain the study objectives [34]. The study employed a multimethod research design carried out in four phases. A multimethod research design involves the use of qualitative and quantitative designs in which each of them is a complete study on their own but together contribute to the components of the entire project [35, 36]. Multimethod designs can be implemented concurrently or sequentially [37]. In this study, a sequential multimethod design was employed as a scoping literature review, a quantitative study and a qualitative study were conducted sequentially as independent complete studies. The lessons learnt based on the findings from these three studies (Table 1) were applied in the development of the framework which was evaluated in the final phase.

### Phase I

The objective of phase I was to conduct scoping review to determine practices that facilitate undergraduate clinical nursing education. An electronic search was conducted in ProQuest (Nursing and Allied Health), CINAHL, ERIC, and PubMed (Nursing Journals). The search was done using keywords: clinical nursing education, student nurse and undergraduate. The articles were screened to identify those that presented key findings on facilitators of clinical nursing education in undergraduate nursing programme.

### Phase II

The objective of phase II was to conduct a situational analysis of the current clinical education programme used in undergraduate nursing education in Ghana. A survey and a qualitative study were conducted to provide this evidence. The survey which was developed for this study assessed the perceptions of preceptors and university nursing students on the quality of clinical nursing education and have been published elsewhere [38]. The preceptors who participated in the quantitative study were registered nurses with at least a diploma in nursing and working in a district, regional or tertiary hospital. Nursing students who were in their second, third and fourth year were recruited for the quantitative study. The qualitative study which was also developed for this study and is published elsewhere explored the perspective of key informants including lecturers, clinical coordinators, unit managers and nurse managers on how the current state of clinical nursing education can be improved [39].

### Phase III

The objective of phase III was to develop the framework for clinical nursing education applying the evidence gathered from phases I and II. The lessons learnt from the findings of phase I and phase II served as data matrix for the development of the framework. The development of the framework was an iterative process in consultation with three nursing education experts.

The Model for Clinical Education developed by South African Nursing Education Stakeholders guided the framework development. Although the model was developed in South Africa it was appropriate for this study because it indicates how the stakeholders in nursing education could work together to improve clinical nursing education. The model also recognises the application of Kolb’s experiential learning theory and Bandura social learning theory in providing conceptual basis for effective teaching and learning of skills. The major organizational structure of the model emphasis the need for experiential learning of clinical skills through effective preceptorship, appointment of a clinical placement co-ordinator, ensuring clinical supervision, ensuring Positive Practice Environment (PPE) and the need for nurse educators to remain clinically competent and take part in clinical supervision [40]. This model leans on the four stages of the Kolb’s experiential learning theory which include concrete learning, reflective observation, abstract conceptualisation and active experimentation. Experiential learning promotes the development of professional skills and knowledge through hands on experience and building of confidence. Also, ensuring a positive learning environment is an essential aspect of promoting effective clinical nursing education and this is consistent with Bandura Social Learning Theory. Bandura social learning theory suggests that most human behaviour are learned observationally through behaviour modelling [41]. Having a Positive Practice Environment can therefore influence skills learning of students.

The major components of the Model for Clinical Education include the Nursing Education Institutions (NEIs), Service Setting, Nursing Students and the Nursing Council. These components served as the priori codes under which the lessons learnt from the findings of the studies in Phases I and II were categorised as shown in Table 1.

Table 1 then served as the data matrix which was iteratively synthesized to develop the framework. The lessons learnt from Phases I and II, displayed in Table 1 were assessed to identify lessons that were common to all the components. The common lessons were coded and categorised into thematic areas based on how they were related. The coding and categorization of the lessons learnt were done in consultation with nursing education experts. The thematic areas were considered as the main constructs of the resulting framework. The presentation of the model and the discussion under each thematic area was done in relation to existing literature.

### Ethical considerations

Ethical Clearance was obtained from the Human Research Ethics Committee (Medical), University of the Witwatersrand (M190807) and Ghana Health Service Ethics Committee (GHS-ERC 007/09/19) for the conduct of the qualitative study and quantitative survey. All the information collected was reported as aggregated data with no reference to any participant. Informed consent was obtained from all participants in the various phases of the study before their participation. The participants were free to withdraw from the study at any time without any negative repercussion.

### Rigour

#### The scoping review

Levac, Colquhoun and O’Brien [42] indicated that consultation adds methodological rigor to the study. The purpose of consultation in this study was to authenticate the findings. Studies were retrieved and selected independently by the first author and a postdoctoral fellow who had several discussion to reach consensus. The study supervisors ratified the findings.

#### The qualitative study

Morse et al. [43]indicated that rigour provides a means of ensuring that the research conducted is not fictional and worth adding to the body of knowledge. The four criteria for ensuring the rigour of a qualitative study which include credibility, dependability, confirmability and transferability were ensured.

#### The survey

Validity is the degree to which an instrument measures what it is supposed to measure while reliability is consistency with which an instrument produces results (Polit & Beck, 2010). The instrument for data collection was adopted, rescaled and pretested. In the survey, the rescaled instrument had an overall Cronbach alpha of 0.925 for preceptors and 0.872 for students.

#### The framework development

The triangulation of the findings from the scoping review, survey and qualitative study, and consultation with the three nursing education experts all contributed to the face and content validity of the developed framework. An implementation plan was developed for the developed framework. Using a Delphi technique clinical experts were invited to evaluate the implementation plan of the framework for feasibility and relevance in the Ghanaian context.

## Development of the framework

**Fig. 1:**
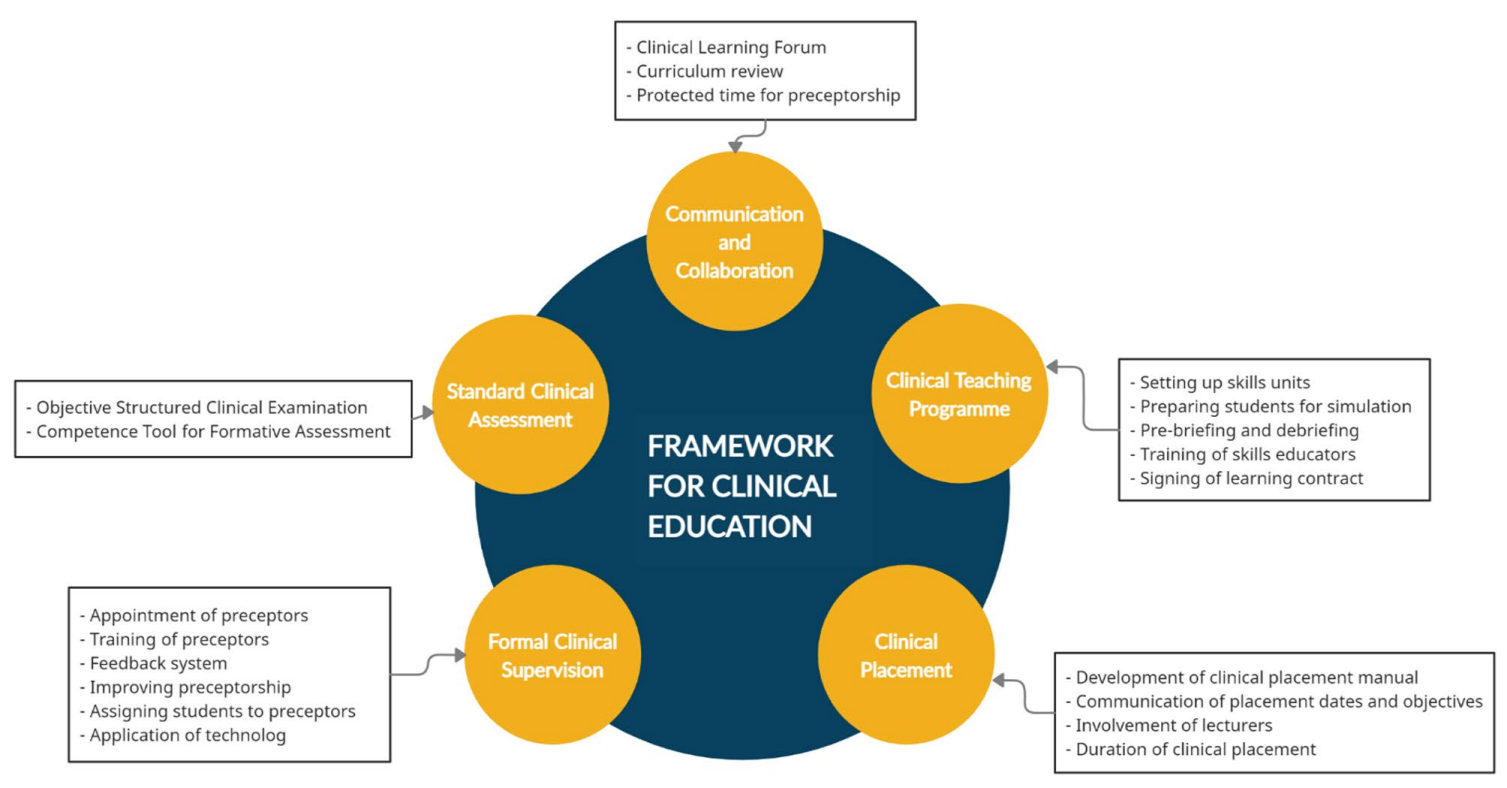
**Framework for Clinical Nursing Education** (Author’s creation)

The goal of the framework (Fig. 1) was to identify and integrate the actions of the various stakeholders to ensure effective clinical nursing education. The framework proposes that to ensure quality clinical nursing education there is the need for NEIs to implement innovative and cost-effective clinical teaching methods, a well-structured clinical placement and a formal supervision system. The service setting which is an integral part of the framework would contribute to improving clinical education through adequate preparation and support of students during clinical placement. The framework (Fig. 1) also indicates that the provision of adequate resources, effective communication and collaboration between NEIs and service settings is crucial in improving the quality of clinical education. The students are the recipients of clinical education and of the activities enshrined in the framework. The students therefore have an important role to ensure that they are fully committed to becoming competent and confident registered nurses on graduation.

**Table 1:** Identification of Lessons Learnt from Phase I and Phase II

Clinical Education Model	Lessons Learnt from Scoping Review	Lessons Learnt from Survey	Lessons Learnt from Qualitative Findings	Common Lessons Learnt from Phase I and Phase II
**Support from NEI**	• A training programme should be developed for preceptors • NEIs should collaborate with clinical institutions in drawing clinical curriculum • Utilise videos in teaching nursing skills • NEIs should improve the application of stimulation • Put students in groups for skills laboratory training • Duration of clinical placement should be consistent with the curriculum as stipulated by Nursing and Midwifery Council of Ghana. • Electronic feedback platform should be used for monitoring students while they are in clinical placement area • Objective Structured Clinical Examination (OSCE) should be used in assessing students • Competence assessment tools are effective in monitoring students progress in skills development	• Pre-publish placement dates to students and clinical facilities should prepare a rotational plan for NEIs to avoid overcrowding. • Ensure adequate duration of clinical placement according to the standard stipulated by Nursing and Midwifery Council of Ghana. • Lecturers should visit the clinical site for supervision • Lecturers should be involved in clinical facilitation • Lecturers should introduce students to “self-directed learning” in the classroom • Communicate clinical placement objectives with clinical facilities before clinical placement • Provide skills laboratories with adequate space and materials • Engage staff for clinical teaching of students in the skills laboratory • Involve preceptors in the development of clinical assessment tools • Transport students to and from the clinical area promptly.	• Intake of students should be according to lecture hall space, facilities for skills training and number of lecturers • There should be a formative assessment of students during clinical placement by preceptors • Appoint adequate number of lecturers for classroom and skills laboratories teaching • NEIs should recruit and train registered nurses to assume the role of preceptors. • NEIs should train simulated patients • There should be capacity building workshops for assessors • Lecturers should take part in clinical supervision • There should effective transportation system • There should be adequate duration for clinical placement as stipulated by Nursing and Midwifery Council of council • Intra-semester clinical placement should be in “block” • NEIs should be adequately equipped skills laboratory	• Improving teaching and learning of clinical skills • communication and collaboration between NEI and service setting • clinical supervision of students • Planning of clinical placement for students • Ensuring effective clinical assessment
**Support from service setting**	• There should be constructive feedback from clinical faculty (nurses, preceptors and clinical supervisors) • Provide material resources for nursing procedures • The clinical facilities should employ adequate number of registered nurses • Steps should be taken to enlist the support of nurses and clinical supervisors in clinical teaching of students • There should be effective communication with student • There should be pre-briefing and debriefing sessions with students during clinical placement • Preceptors should have characteristics such as effective communication skills, coaching skills and role modelling. • Peer learning i.e. pairing of students from same level of study for the same shift during clinical placement should be encouraged	• Clinical facilities should develop a clinical manual containing all rules regarding clinical practice and procedures • Preceptors should clarify the learning needs of students • The clinical facilities provide adequate equipment for demonstrations and return demonstration • Preceptors should have effective communication with students • Preceptors should develop a remedial plan for students who are unable to gain competence in a particular skill • Sign learning contracts with students to guide skills teaching and achievement of competences.	• Effective communication with academic institutions of clinical learning outcomes of student • Rotational plan for various academic institutions that send students for clinical placement • Engaging more registered nurses to support students in clinical learning • Provision of material resources for students to learn skills • Encouraging nurses to initiate personal strategies to enhance clinical learning at the ward level • Peer learning i.e. the pairing of students who are at the same level of study but in different schools to enable them to learn from each other • Preceptors should conduct formative assessment • Organize clinical meetings with students during clinical placement	• Preparation to receive students • Communication between service settings and NEI, and service setting and students • Support of Students during clinical placement • Provide material and human resources • Clinical assessment
• **Student in clinical for role taking practice**	• Students should indicate readiness for clinical learning through observation, listening and asking questions • Students should attend pre-briefing and debriefing sessions during clinical placement • Students should be self-confident and motivated	• Students should accept constructive criticism and make efforts to learn • Sign a performance contract before clinical assessment • Students should communicate effectively with clinical staff • Students should avail themselves for clinical assessment	• Students should attend clinical conferences and ask questions • Students should seek counselling when they have problems in the placement area • Students should respect laid down rules of clinical placement	• Attendance • Positive mindset • Commitment
**Support from Nursing Council**	• NM&C should monitor NEIs through scheduled visits	• Assist academic institutions set-up skills laboratories that have essential equipment and materials	• NM&C should set-up an accreditation system for clinical facilities • NM&C should make recommendations for student intake based on the availability of facilities • Monitor NEIs to provide adequately equipped skills laboratory • NM&C should improve conduct of Licensing Examination	• Monitoring to ensure that NEIs meet and maintain a minimum standard • Advice to the Minister of Health • Clinical assessment of students

### Thematic areas of the framework

The resulting framework as presented (Fig. 1) consists of five thematic areas which include communication and collaboration, clinical teaching, formal clinical supervision, clinical placement system and standard clinical assessment. These thematic areas work together to contribute to an effective clinical nursing education.

### Communication and collaboration

This aspect of the framework indicates that collaboration and communication between the NEIs and the health facilities is necessary for improving clinical nursing education. Communication and collaboration were seen as important factors in the formation of a clinical learning forum, reviewing and developing the curriculum for clinical training, information flow and finding protected time for preceptors.

#### Clinical learning forum (CLF)

CLF is a set of committees that will work in a coordinated manner to ensure effective clinical nursing education is an essential aspect of communication and collaboration. The formation of a clinical learning forum consists of representatives from NEIs and the service setting. Academic-clinical partnership bridges the gap between education and practice and brings stakeholders closer together [44, 45]. Evidence gathered in this study indicates current challenges with regard to communication between students and both the NEI and the clinical facilities, the researcher therefore recommends students be included the CLF.

The roles of members of this forum are designed to ensure that students are placed appropriately and that all clinical learning takes place in an optimal manner. The forum oversees all the activities identified in the framework. The CLF should consist of subcommittees specifically responsible for clinical placement, clinical teaching, formal clinical supervision and clinical assessment. The CLF will need to have a constitution to guide their work. The CLF should have scheduled meetings, review of subcommittee reports and maintain effective communication with Nursing and Midwifery Council.

#### Curriculum review

The framework indicates that when clinical nurses and preceptors take part in preparing the clinical education curriculum it will enable them to make contributions based on their clinical experience. Also, when preceptors are involved in review of the curriculum, they can determine areas that the preceptors themselves will need further training to enable them to assist students with skills learning. Academic-clinical collaboration is recommended to review and draw up clinical education curricula [46].

The absence of a practical workbook or manual containing the steps and rationale for nursing procedures causes incongruence among clinical supervisors [47]. There is the need for NEIs to spearhead an academic-clinical collaboration to develop a clinical workbook that reflects current standard guidelines for the performance of nursing procedures.

#### Protected time for preceptorship

Preceptors are experienced nurses who contribute to the professional development of nursing students [48, 49]. Preceptors create a conducive clinical learning environment and guide students to achieve their placement objectives. In Ghana, preceptors are nurses employed by the clinical facilities but are engaged by NEIs to do clinical teaching of students in addition to their responsibilities as nurses. The current arrangement does not allow the preceptors adequate time to support students during clinical placement. The current framework therefore recommends the need for NEIs and clinical facilities to plan a protected time for preceptors to engage in preceptorship when students are in the clinical facility for placement.

### Clinical teaching

The framework established that effective clinical teaching is necessary in building the professional skills of nursing students. To ensure efficient skills teaching there is the need to set up skills units, prepare students for simulation, instituting the pre-briefing and debriefing process, assigning staff for skills education and training skills educators. Other innovative teaching strategies such as peer-learning and signing of learning contracts were included in the framework.

#### Setting up skills units

Setting up a skills unit allows students to practice skills in an environment that mimics the clinical setting was established as an essential need effective skills teaching and learning. The skills unit gives students a platform to practice and gain clinical skills without fear of causing harm to human life [50]. The global standards for effective nursing education require the use of innovative methods such as simulation for initial nursing training [51]. NEIs should therefore identify space and work on a budget towards setting up a skills laboratory as a basic requirement in providing a simulated environment for skills training of students.

#### Preparing students for simulation

Simulation-based learning is an effective approach that enables students to practice clinical skills, solve problems and make decisions [52]. Students should be introduced to the various simulation strategies such as role-play, standardised patients, computer-based simulation, virtual reality and high fidelity. Preparation of students for simulation vary and could include reviewing procedures, writing pre-quizzes, watching manikin videos and signing confidential agreements [53]. Posting of videos of nursing procedures for students to watch before training sessions is a viable strategy recommended to allow the students to develop confidence towards performing the procedures [54].

#### Pre-briefing and debriefing

The findings from phase I of this study revealed that pre-briefing and debriefing enhances clinical teaching and learning. Pre-briefing positively influences students’ competency performance and clinical judgment [55]. Pre-briefing involves a face-to-face discussion between students and skills educators. During the pre-briefing, the staff gives guidance based on the placement objectives. The specific procedure that will be practiced vis-à-vis the available equipment or material resources to ensure that students achieve clinical placement objectives is discussed during pre-briefing.

Debriefing will enable preceptors to give feedback to students on their performance and make clarifications where necessary. Debriefing provides an opportunity for preceptors and students to improve skills teaching and learning through healthy reflection and discussion of clinical events.

#### Assigning staff for skills education

Skills educators are lecturers or staff of NEIs assigned to teach skills in the skills laboratory. Skills educators also play an important role in clinical teaching by accompanying students to the clinical placement area. The findings of phase II of this study established that there are currently no staff placed to manage the skills laboratory at the study site. NEIs need to engage staff whose main responsibility will be to teach clinical skills in the skills laboratory. Assigning academic lecturers for skills teaching in the skills laboratory will ensure that students get the required support in skills learning. In the Ghanaian context, NEIs with inadequate number of lecturers could explore the option of engaging senior research assistants for skills teaching upon building their capacity. Senior research assistants are staff with at least a bachelor’s degree employed by NEIs to assists lecturers with teaching and learning activities.

#### Training of skills educators

The knowledge and skills of the skills educators is an essential factor in the clinical training of students. The NEIs need to develop Continuous Professional Development (CPD) programmes with corresponding CPD points to build the capacity of skills educators to support students. Skills educators need training on proper questioning technique, giving cues and giving feedback to students during skills learning [48]. Skills educators’ ability to ask appropriate questions will help students to think critically and broaden the students’ scope on the skill that they are performing. The skills educator needs to listen, read facial expressions and frame questions to learners level of understanding [56]. Verbal and non-verbal cues such as nodding, thumbs-up and smiling are powerful in encouraging students during the performance of skills. Constructive feedback should start and end on a positive note focusing on the performance of the student and not the character [48]. Also, the skills educator should be able to establish rapport and motivate students to participate in skills learning activities [52].

#### Learning contract

Students come to the ward with placement objectives that they need to achieve. Learning contracts promote self-directed learning [57]. When there is a learning contract students become active participants in their learning by making contributions to what is to be learnt and how it should be learnt [58]. In self-directed learning students identify resources and take action to achieve their learning needs [59]. The framework recommends that learning contracts should therefore be implemented to during clinical placement. However, there must be an educational preparation of students on the concept of self-directed learning and learning contract. The preparation of the students on importance and implementation of learning contracts could be factored into the orientation programme for students during clinical placement.

### Clinical placement system

Clinical placement is an important activity in the training of nursing students. The areas of clinical placement that emanated from situational analysis and are captured this framework include the need for development of a clinical placement policy manual, communication of placement dates/objectives, involvement of lecturers and ensuring that duration of clinical placement is consistent with recommendations of the Nursing and Midwifery Council of Ghana.

#### Clinical placement manual

There is a need for development of a clinical placement policy manual for all the clinical sites. The manual should contain duties and responsibilities of students, lecturers, preceptors, nurse managers, unit managers and registered nurses in relation clinical placement. The manual will also contain ward policies. The students should be taken through the manual during an orientation programme at the commencement of clinical placement.

#### Placement dates and objectives

The situational analysis established that clinical facilities receive students from various NEIs at a time hence negotiating placement dates will enable scheduling to avoid overcrowding of students. Clinical placement dates and objectives should be communicated at least 2 months ahead to the clinical facilities to prepare for the placement. The clinical placement coordinators facilitate the preparation for clinical placement of students by informing nurse managers, clinical preceptors and ward nurses about placement dates [60]. The NEIs may also need to discuss with clinical facilities regarding competencies the students are suppose to acquire. This will help determine where students should be placed and the support that is required to achieve the clinical placement objectives.

#### Involvement of lecturers

When lecturers are involved in clinical supervision it will enable them to facilitate clinical skills learning by linking theory to practice [61]. The involvement of lecturers in clinical supervision will enable them to guide students in the learning of skills, assess students’ progress and supervise preceptors. The presence of lecturers in the clinical environment will enhance interaction between lecturers and preceptors in the interest of improving student support. Clinical supervision should therefore be factored in as a responsibility of nursing lecturers. Where lack of lecturers is a major challenge, senior research assistants could be engaged to act as clinical supervisors.

#### Duration of clinical placement

Nursing education consists of a blend of theory and practice which may be given an equal amount of time [62]. The duration of clinical placement is a very important factor because nursing is a practice-based profession. Spending a longer duration of time in the clinical placement area is associated with higher exposure to clinical skills learning [61, 63]. Findings from both phase I and phase II all indicated that clinical exposure given to the students was inadequate. The curriculum for undergraduate nursing training stipulates the duration of clinical placement in accordance with recommendations from Nursing and Midwifery Council of Ghana. The Nursing and Midwifery Council of Ghana curriculum for training of registered general nurses stipulates that nursing students in the first, second and third year should spend 432 h, 624 h and 576 h in the clinical placement [64]. These clinical placement hours are to be achieved through both intra-semester and inter-semester clinical placement. NEIs should therefore ensure that clinical placement duration reflects the stated hours in the curriculum.

The qualitative study in phase II established that another factor that cut down on the extent of clinical exposure was late reporting on duty and early departure of students during clinical placement. To overcome this, NEIs need to ensure that bus drivers and students understand the need for early reporting and to have a monitoring system put in place to ensure adherence to the allocated clinical hours. Administrative issues such as availability of buses, maintenance and fuel supply should all be addressed by NEIs to ensure effectiveness.

### Formal clinical supervision system

Lessons from phase I and II of the current study all suggested that clinical supervision of students during clinical placement is a major element of improving the clinical education of students. The elements of clinical supervision necessary for effective clinical nursing education include the appointment of preceptors, training of preceptors, feedback on preceptorship, improving preceptorship, assigning preceptors to students and application of technology in clinical supervision.

#### Appointment of preceptors

Preceptors play a very important role in assisting students with the learning of clinical skills during clinical placement [10, 65–67]. In the Ghanaian context, preceptors may not have formal training but are selected based on their experience as nurses and their willingness to support students. The present framework indicates the need for clinical facilities to collaborate with NEIs to identify and appoint nurses for the role of preceptorship. The group of key informants to select potential preceptors should include lecturers who teach or coordinate clinical nursing, nurse managers, unit managers and clinical placement coordinators. The NEIs determine the content of clinical education and the clinical facilities supervise students to acquire the stipulated competencies hence the need for collaboration in selecting potential preceptors. Prospective preceptors should be professional nurses who can communicate effectively, uphold the ethics of nursing and have a desire for professional growth [68]. The preceptor should have the willingness, competence and good communication skills [69].

#### Training of preceptors

A best practice workshop should be developed to train appointed nurses to assume the role of preceptors. The training of preceptors should cover areas such as principles of clinical teaching, learning and assessment in the adult context, roles of the preceptor, and management of student-preceptor interaction [48]. Also, the training should include the application of knowledge, skills and attributes in clinical teaching and learning, how to initiate interest in learning using creative strategies, and how to monitor and evaluate tailor-made programmes [70]. Dates for training preceptors should be agreed upon with authorities of the clinical facilities to enable the selected staff to participate.

#### Feedback system

NEIs need feedback from preceptors and students to be able to evaluate the effectiveness of the preceptorship implemented for students [71]. Feedback could be collected through questionnaires or an online feedback form. The NEIs should adopt at least one of these strategies to evaluate effectiveness of clinical placement each semester. Changes implemented based on feedback strengthens the collaboration between the two institutions and improves students’ learning experiences.

#### Improving preceptorship

Effective preceptorship will ensure that students receive adequate support during clinical placement. One of the barriers facing the effective implementation of preceptorship in Ghana is the lack of motivation for preceptors by NEIs [65]. Hence there is the need to map out strategies to motivate preceptors. Preceptors could be motivated by appointing them as examiners for clinical assessment of students. Other incentives that could be given to preceptors include awarding CPD points for the preceptorship they provide students and formal recognition as adjunct faculty through appointment letters. Preceptors could also be given access to NEIs facilities such as the University library and online resources. Instituting a feedback system to assess the perspective of preceptors on the barriers, motivating factors and how to improve preceptorship should also be considered.

#### Assigning students to preceptors

The number of preceptors to be trained should reflect the number of students that need supervision. Several recommendations on the preceptor-student ratios have been made which include 1:6 [72, 73], 1: 15 [62] and 1:15–20 [49]. Considering all these recommendations provided by published literature will be necessary when assigning students to preceptors.

#### Application of technology

The application of technology in monitoring students during clinical placement is also a viable strategy that could be applied to supporting students [74]. An electronic platform that requires students to give feedback on daily basis is an effective means of monitoring students’ progress in clinical placement. The feedback received by NEIs can help determine if the students are receiving adequate support from preceptors and areas that skills training is lacking or needs improvement.

### Standard clinical assessment

Clinical assessment is a strategy for assessing students’ progress in clinical skills acquisition. It also assesses the students’ competence, the need for student remediation and provides students with feedback. There is the need to develop consistent and objective assessment methods [75]. To improve clinical assessment, the framework established the need to review and update Objective Structured Clinical Examination (OSCE) assessment tools, and train clinical assessors and simulated patients. Preceptors also require training in the application of assessment tools for formative assessment.

#### Objective structured clinical examination (OSCE)

Objective Structured Clinical Examination (OSCE) is considered an effective tool in the clinical assessment of students [76]. Findings from the qualitative study indicated that the OSCE is currently used in the clinical assessment of students in the skills laboratory but this requires improvement. Experienced examiners, educational experts and external moderators should be involved in the development and updating of the assessment tools and training of the clinical assessors. To make the clinical assessment in the skills laboratory mimic the clinical environment there is the need to also train the simulated patients.

Pre-examination conferences should be done to discuss the clinical assessment tools and assessment criteria to be used. All examiners need to be briefed about the objectives of the exam, and what level of skills competency is expected from the students. Post-examination conferences should be organized to discuss students’ strengths and weaknesses and make recommendations for improvement. A feedback form should also be designed for students to comment on the conduct of the assessment and what in their view should be continued or needs improvement.

#### Competence tool for formative assessment

Authentic formative assessment involves assessing the performance of students in actual role-taking in the face of available resources and time [77]. The formative assessment focuses on the students’ behaviour and performance of clinical skills and not on the personal character of the students [78]. Formative assessment allows students to receive feedback on their performance. Preceptors should be trained in the application of, and assist in the development of competence tools for formative assessment. The formative assessment will help in identifying areas that students have developed competence and areas that they need more support. Where formative assessment reveals that a student is unable to gain competence, a remedial plan should be instituted to enable such a student to gain the needed competence. The remedial plan may involve allocating more clinical placement hours, and giving additional academic and supervisory support. The remedial plan could also involve clinical skills practice in the skills laboratory with a clinical facilitation.

### Framework implementation plan

The final step of developing the draft framework was to develop an implementation plan for the framework (Table S1). The implementation plan consisted of five standards that were consistent with the five thematic areas of the framework. The implementation plan had three phases which included the immediate, intermediate and final phase. The immediate phase consisted of actions that were implementable in the first six months, the intermediate phase consisted of actions implementable beyond the first six months but within 2 years and the final phase consisted of the next 3 years.

### Evaluation of the implementation plan

The implementation plan was evaluated using a Delphi technique. In phase one of the Delphi, responses indicated that quantitatively there was consensus on all items in the questionnaire. However, the qualitative comments indicated that there were areas that the respondents were not satisfied. Phase two of the Delphi was conducted as a follow-up based on the comments indicating dissatisfaction in phase one. Phase two allowed for modifications and additions to the final implementation plan of the framework. The Delphi survey have not been published as at now.

### Strengths of the study

The overall aim of the present framework and some aspects of the structure are similar to that of the model for clinical education by the nurse educators group of south Africa [46]. However, the organisation structure of the present framework and details looks different.

This is the first study focusing on the development of a framework for clinical education of undergraduate nursing students in Ghana. The framework will provide a guide to enhance the clinical education component of undergraduate nursing education. The framework captures the essential aspects of clinical education in nursing which can impact clinical skills teaching and learning when implemented.

The involvement of nursing students, preceptors, nurse educators and nurse managers in the situational analysis allowed for assessment of the current clinical education from a multidimensional perspective. This also makes the framework more practical as all these participants are stakeholders in clinical education whose essential experiences have implications for clinical education in nursing.

### Limitations of the study

The situational analysis was conducted among participants recruited from one public university and three clinical facilities in Northern Ghana. This may affect the generalizability of the findings of the situational analysis which was integral in providing data for the framework development. However, lecturers from other universities in southern Ghana, and the Nursing and Midwifery Council of Ghana participated in the Delphi survey where they assessed the framework and made inputs to make the framework more applicable in the wider Ghanaian context.

The situational analysis also provided context specific information on how clinical education could be improved but this could be viewed to be limited in scope. However, the use of the scoping review allowed for a wider assessment of how clinical education in nursing could be improved applying lessons from different context.

## Conclusion

The evidenced-based framework developed in this study consisted of five standards which include communication and collaboration, clinical teaching programme, formal clinical supervision, clinical placement and a standard clinical assessment. The framework developed indicates that clinical nursing education can be improved through the identification and integration of the roles of the stakeholders in improving each of these standards. The Clinical Learning Forum (CLF) proposed in the framework is fundamental in implementing an evidence-based framework to advance clinical education. The formation of a CLF will consist of subcommittees with membership drawn from all stakeholder institutions. The roles of the CLF subcommittees will essentially implement evidence gathered and spelled out in the framework. The effectiveness of the CLF subcommittees will translate into the implementation of a clinical teaching programme, formal clinical supervision, clinical placement and a standard clinical assessment system proposed in the implementation plan of the framework.

### Electronic supplementary material

Below is the link to the electronic supplementary material.

Supplementary Material 1

## Data Availability

The data that supports the current study are available with the corresponding author upon reasonable request.
