# Supplementary material for: The development of a framework for clinical education programme of undergraduate nursing students in Ghana
Source: BMC Nurs. 2024 Apr 23;23:263. doi: 10.1186/s12912-024-01915-y (PMC11036577; doi:10.1186/s12912-024-01915-y)
Supplement: Supplementary file 1 — Supplementary Material 1 [file 12912_2024_1915_MOESM1_ESM.docx]

**Table S1: Implementation Plan of the Clinical Nursing Education Framework**

| **Standard** | **Initial Phase**  **First 6 months** | **SH** | **Intermediate Phase**  **In the next 2^nd^ year** | **SH** | **Final Phase**  **3^rd^, 4^th^ & 5^th^ year** | **SH** |
| --- | --- | --- | --- | --- | --- | --- |
| C**ommunication and collaboration between stakeholders** provide for seamless preparation of students in the classroom and clinical areas | - Constitute a Clinical Learning Forum (CLF) with representation from NS, NEI and SS to monitor implementation of the framework - Write constitution for a Clinical Learning Forum (CLF) - The CLF should include sub-committees on the clinical teaching programme, the formal system of clinical supervision, clinical placements and clinical assessment - The representatives should meet to establish ground rules and plans | NS  NEI  SS  NS  NEI  SS  NS  NEI  SS  NS  NEI  SS | - Meet regularly and function as a CPF according to constitution of the CPF - Appoint members onto sub-committees - Review quarterly reports submitted by sub-committees - Maintain communication with the Nursing and Midwifery Council - Provide feedback of NM&C activities and changes to all stakeholders | NS  NEI  SS  NEI  SS  NEI  SS  NEI  NEI | - Review roles and functioning of CLF and amend as required - Continue regular meetings and activities | NS  NEI  SS  NS  NEI  SS |
| **Clinical teaching** **programme** will provide cost-effective, innovative and relevant education to prepare students for their future roles as professional nurses | - Clarify the learning needs of the Student before clinical placement or skills laboratory sessions - Pre-test students a week prior to placement - Plan a pre-briefing and debriefing for clinical teaching - Identify an area in the department to be used as a skills unit - Work out a budget for requirements for the skills unit - Video skills that will be taught in the skills lab should be posted on the students learning platform for pre-skills preparation - Remind students at least 4 weeks to clinical placement | NEI  SS  NEI  NS  NEI  SS  NEI  NEI  NEI  NEI | - Sign learning contracts with students - Introduce students to self-directed learning - Purchase equipment for skills laboratory - Provide low-cost simulation aids - Develop the designated area as a skills unit - Orientate students to effective use of skills unit - Provide specialised training for lecturers on high and low fidelity simulation and innovative skills teaching methods - Purchase audio visual equipment for the real-time transmission of scenario simulations and debriefing to students not in the skills unit. - Plan for embedding small group clinical skills teaching in the curricula. | NEI  SS  NS  NEI  SS  NEI  NEI  NEI  NEI  NEI  NEI  NEI | - Purchase additional simulation equipment - Plan a yearly evaluation of the effectiveness of steps taken to improve clinical teaching - Implement changes as required | NEI  NEI  SS |
| **A formal system of clinical supervision** provides structure and support to students to enhance translation of theory into practice | - Develop a budget and motivate for funding for the engagement of preceptors - Develop a memorandum of understanding between the service settings and the NEI on the selection, appointment and training of preceptors for student supervision. - Determine the position and responsibilities of the existing clinical facilitators (registered nurses) in relation to the preceptors and the nursing students - Determine the position and responsibilities of the nurse educators in relation to clinical supervision. - Design a best practice workshop on clinical supervision skills. - Develop an electronic monitoring system of students during placements | NEI  NEI, SS  NS  NEI  SS  NEI  SS  NEI | - Appoint preceptors - Training dates should be agreed upon with authorities of the clinical facilities to enable the selected staff to attend. - Expose all preceptors, clinical facilitators and nurse educators to the best practice workshop on clinical supervisory skills - Allocate students to preceptors that have participated in the supervision training course for ongoing clinical supervision - Develop a feedback system for both preceptors and students to discuss the level of supervision provided to the student. - Improve preceptorship by implementing lessons learnt from feedback system - Train users on how to use the electronic platform for clinical supervision | NEI  SS  NEI  SS  NEI SS  NEI  SS  NEI  NEI, SS  NEI | - Review the attendance rate and feedback from the attendees at the workshops. - Conduct a research study on the translation into practice of the supervisory skills taught in the workshop - Review the success (level of attendance, and student feedback) of the supervision training program and refine as required. | NEI  NEI  NEI |
| The **clinical placement** system is structured to facilitate optimal exposure to practice and appropriate student assessment | - Develop a clinical placement policy manual containing ward policies, duties and responsibilities of students, lecturers, preceptors and clinical facilitators. - Negotiate placement dates with the service setting | SS  NEI  NEI  SS | - Make dates available to all stakeholders at least 2 months prior to placement - Schedule regular dates and times, in the academic year for lecturers from the NEIs to be present in the clinical areas to assist with supervision and clinical teaching - Send specific clinical placement objectives to clinical site and students who are attending the specific clinical site a month prior to the placement | NEI  NEI  NEI | - Review clinical placement manual to evaluate relevance/alignment to student’s clinical needs. - Review success of electronic monitoring system - Make changes as required | NS  NEI  SS  NEI  NEI  SS |
| A **standard clinical assessment** system will ensure effective monitoring of the skills development of students | - Constitute an expert committee consisting of experienced examiners, educational experts and external moderators to review and update clinical assessments tools - Expert committee should design a training programme for assessors and simulated patients | NEI  NEI | - Organise a training for OSCE assessors and simulated patients’ clinical assessment - Institute pre- and post-examination conferences - Train preceptors on the use of competence tools for formative assessment - Develop remedial plans for students who are unable to achieve competence during clinical placement - Move clinical assessment of students in the second year and beyond to the clinical area | NEI  NEI  NEI  NEI  SS  NEI | - Schedule a yearly refresher training of simulated patients and assessors - Evaluate the effectiveness of using competence tool for formative assessment - Review and update clinical assessment tools every two years - Train new assessors and simulated patients prior to clinical assessment each time | NEI  NEI  NEI  NEI |

Key: SH = Stakeholder NEI = Nursing Education Institution; NM&C = Statutory council; SS = Service setting; NS= Nursing students
